# DNA-Templated Silver Nanoclusters Demonstrate Potent Antimicrobial Activity Against the Clinically Relevant Pathogens, *Neisseria meningitidis* and *Streptococcus pneumoniae*

**DOI:** 10.1021/acsabm.5c02143

**Published:** 2026-02-18

**Authors:** Krishna J. Majithia, Elizabeth Skelly, Kirill A. Afonin, M. Brittany Johnson

**Affiliations:** Department of Biological Sciences, University of North Carolina at Charlotte, Charlotte, North Carolina 28223, United States; Chemistry and Nanoscale Science Program, Department of Chemistry, University of North Carolina at Charlotte, Charlotte, North Carolina 28223, United States; Chemistry and Nanoscale Science Program, Department of Chemistry, University of North Carolina at Charlotte, Charlotte, North Carolina 28223, United States; Department of Biological Sciences, University of North Carolina at Charlotte, Charlotte, North Carolina 28223, United States

**Keywords:** silver nanoclusters, antimicrobial, Neisseria meningitidis, Streptococcus pneumoniae, meningitis

## Abstract

Antimicrobial resistance is growing among the causative agents of bacterial meningitis, *Neisseria meningitidis* and *Streptococcus pneumoniae*, which trigger detrimental neuroinflammatory responses within the central nervous system. Here, we evaluated the antimicrobial potential of DNA-templated and stabilized silver nanoclusters (AgNCs). AgNCs were templated on a single hairpin (HP) or fibrous hairpin structures (HP-F). HP-F provided higher local concentrations of AgNCs, when compared to HP, and exhibited stable physicochemical properties and potent antimicrobial activity. Furthermore, at low silver concentrations, AgNCs restricted bacterial survival and reduced inflammatory responses in microglia without causing cytotoxicity, supporting further development of AgNCs as therapeutics for meningitis.

## Graphical Abstract

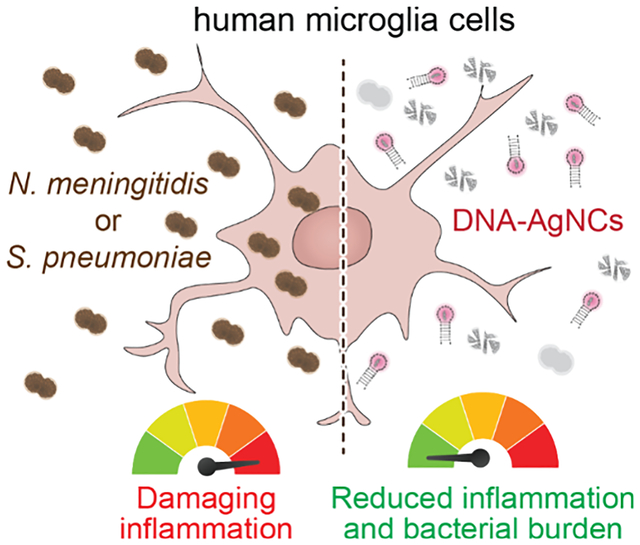

Antimicrobial resistance (AMR) represents one of the greatest global health challenges, threatening the effectiveness of existing antibiotics and complicating the treatment of common infections.^1,2^ The continued rise of multidrug-resistant bacterial strains underscores the urgent need for new antimicrobial strategies that are both effective and adaptable. Among the pathogens of particular concern are *Neisseria meningitidis* and *Streptococcus pneumoniae*, two leading causes of bacterial meningitis worldwide.^3–7^ Bacterial meningitis remains a major cause of neurological morbidity and mortality, with an estimated 2.51 million cases and over 230,000 deaths reported globally.^8^ During infection, bacterial invasion of the meninges triggers a robust inflammatory response within the central nervous system (CNS), resulting in disruption of the blood–brain barrier, neuronal injury, and potentially irreversible neurological damage.^9–11^ Given the relatively enclosed intracranial space and the rapid progression of inflammation during infection, timely treatment is critical, and delays can lead to detrimental outcomes.

Historically, both *N. meningitidis* and *S. pneumoniae* have been treated with *β*-lactams and macrolides; however, rising antimicrobial resistance poses a significant challenge to treatment.^12–15^ *N. meningitidis* has shown a concerning rise in resistance since 2019, particularly in serogroup Y strains that can disseminate resistance genes leading to reduced susceptibility to penicillin, ciprofloxacin, and rifampicin.^16^ For *S. pneumoniae*, resistance to penicillin, macrolides, and fluoroquinolones has continued to emerge globally and is driven mainly by alterations in penicillin-binding proteins and efflux pump mechanisms.^17^ These developments threaten the effectiveness of current therapeutic regimens. In response, new prevention and treatment guidelines have been issued to address antimicrobial-resistant meningococcal disease. In 2024, the U.S Centers for Disease Control and Prevention updated recommendations to include antimicrobial susceptibility testing for all *N. meningitidis* isolates, alternative chemoprophylaxis regimens for ciprofloxacin-resistant strains, and increased national surveillance of resistant clones.^18^ These measures reflect growing recognition that the AMR of both *N. meningitidis* and *S. pneumoniae* poses an urgent public health threat.

Given this context, there is an increasing need to explore nontraditional antimicrobial strategies that circumvent resistance mechanisms. One promising strategy involves the use of nanomaterials with inherent antimicrobial properties. Interestingly, nanosilver has been recognized for its broad-spectrum antibacterial activity and has been increasingly applied in medical devices and wound coatings.^19,20^ Silver nanoparticles (AgNPs) have been extensively studied and are recognized as potential therapeutics. However, AgNPs have several limitations. Their multistep synthesis typically requires specialized equipment and lengthy, time-consuming protocols, often resulting in particles that lack colloidal stability and are polydispersed. Moreover, additional surface modifications are often needed to improve water solubility and reduce aggregation, further limiting scalability and clinical relevance.^21^ Recent advances in nanotechnology have enabled the templating and stabilizing of silver nanoclusters (AgNCs) with nucleic acids (RNA and DNA), generating fluorescent AgNCs that also combine the antimicrobial activity of silver with the programmability of biocompatible nucleic acids.^22–24^ As such, DNA-templated AgNCs offer several advantages, including tunable size and fluorescence properties,^25^ high biocompatibility, batch-to-batch consistency, scalability, and the potential for functionalization.^21^ Additionally, DNA can be linked to other nucleic acid-based constructs, such as nucleic acid nanoparticles used to boost immune responses,^23^ reconfigurable nucleic acids that respond to intracellular cues,^26^ and various targeting moieties^27^. Consequently, this emerging class of nanoscale silver formulations holds great promise as antimicrobial agents with the potential to address therapeutic needs during a broad range of bacterial infections. In this study, we investigated the antimicrobial activity of nanoassemblies made of multiple copies of DNA-templated AgNCs against *N. meningitidis* and *S. pneumoniae*, two major drivers of meningitis, to assess their potential as novel therapeutic agents.

Based on recent studies,^22,28^ we selected a single hairpin (HP) containing a loop of 13 single-stranded cytosines (Figure 1A), as the DNA template for AgNCs with demonstrated strong antimicrobial activity. To increase the local concentration of AgNCs and potentially enhance their antimicrobial efficacy, we decorated these HPs with complementary ssDNA toeholds designed to promote one-pot self-assembly of multiple HPs into fibrous architectures, termed HP-F AgNCs.

The assembly of HP-F structures was first evaluated by 8% nondenaturing polyacrylamide gel electrophoresis (native-PAGE) and compared to free HPs as controls. As expected, HPs migrated as a single discrete band, while HP-F produced a diffuse smear corresponding to a higher molecular weight species (Figure 1B). This pattern indicates heterogeneous assembly of the HP-F of higher-order multimers, with some species more abundant, as shown by the prominent lower band.

Consistent with previous studies,^22^ both HP- and HP-F-templated AgNCs exhibited red fluorescence with emission peaks at 645 and 620 nm, respectively (Figure 1C–D). The peak excitation wavelengths were 565 nm for the HP AgNCs and 545 nm for the HP-F AgNCs. Across a range of excitation wavelengths, HP AgNCs displayed a narrower emission spectrum, whereas HP-F AgNCs showed a higher peak intensity (Figure 1C–D). Both samples have a peak emission in the red portion of visible light, highlighting the potential of these structures for use in bioimaging applications. However, the narrow emission range and greater spectral definition of HP suggest that it may serve as a more precise imaging agent (Figure 1D). After storage at 37 °C for 1 week, the samples lost fluorescence (Figure 1E). The excitation peak for HP AgNCs became 580 nm, and that for HP-F AgNCs became 370 nm. The emission peaks for HP and HP-F AgNCs were 665 and 440 nm, respectively.

In addition, energy-dispersive X-ray spectroscopy (EDS) was conducted to evaluate the number of silver atoms per HP after the formation and purification of HP AgNCs. It was found that 8.59 ± 0.64 atoms of silver are on each hairpin structure (Figure S1).

Due to the antimicrobial activity of these constructs, we investigated if delivery of HP and HP-F AgNCs to glial cells can restrict bacterial burden and consequently reduce damaging inflammatory responses (Figure 1F).

Minimum inhibitory concentration (MIC) and minimum bactericidal concentration (MBC) assays were performed to evaluate the antimicrobial activity of these constructs against *N. meningitidis* and *S. pneumoniae*, with all concentrations reported as silver equivalents. HP AgNCs demonstrated potent, dose-dependent antimicrobial activity, as evidenced by the significant inhibition of bacterial growth and viability (Figure 2A–D). For *N. meningitidis*, the growth was effectively inhibited at the lowest concentration tested (0.8 *μ*M), suggesting a greater sensitivity to HP AgNCs. However, complete inhibition of growth, defined as the MIC, was achieved at a higher concentration of 52 *μ*M silver (Figure 2A). Additionally, the viability of *N. meningitidis* was significantly decreased at 6.5 *μ*M, indicating that lower concentrations primarily exert bacteriostatic rather than bactericidal effects. Complete bactericidal activity against *N. meningitidis*, as defined by the MBC, was observed at 104 *μ*M silver (Figure 2A). These data show that growth is impaired at concentrations below those required for bactericidal activity with the HP. For *S. pneumoniae*, growth inhibition with the HP AgNCs was observed at 1.6 *μ*M and a MIC occurring at 104 *μ*M (Figure 2C). Notably, viability was reduced at 0.8 *μ*M, and the MBC was determined to be 104 *μ*M. (Figure 2C). Collectively, these data indicate that HP AgNCs exert distinct antimicrobial effects on *N. meningitidis* and *S. pneumoniae*.

We next compared the antimicrobial activity between HP and HP-F AgNCs by determining the concentrations required to reduce the bacterial growth and viability of *N. meningitidis* and *S. pneumoniae*. Using the HP-F AgNCs, the level of growth of *N. meningitidis* was significantly reduced at 0.8 *μ*M, with complete inhibition observed at an MIC of 52 *μ*M silver (Figure 2B), similar to the observed effects of the HP AgNCs (Figure 2A). HP-F significantly reduced bacterial viability at 1.6 *μ*M, with complete bactericidal activity achieved at 104 *μ*M silver (Figure 2B). Notably, HP-F AgNCs significantly reduced bacterial viability at a lower concentration compared to HP AgNCs supporting enhanced bactericidal activity. For *S. pneumoniae*, HP-F AgNCs resulted in reduced growth at a lower concentration (0.8 *μ*M) than HP AgNCs (1.6 *μ*M) (Figure 2C), with an MIC of 104 *μ*M (Figure 2D). Additionally, a reduction in *S. pneumoniae* viability was observed at 6.5 *μ*M, with an MBC of 104 *μ*M silver (Figure 2D). Importantly, both HP and HP-F AgNCs maintained comparable antimicrobial activity after 7 days at 37 °C, indicating stability under physiologically relevant conditions (Figure S2A–D).

Consistent with strong antibacterial activity resulting in membrane damage, Live/Dead bacterial staining demonstrated a significant reduction in both *N. meningitidis* and *S. pneumoniae* viability following treatment with HP and HP-F AgNCs compared to untreated controls (*N. meningitidis* HP AgNCs treated 75.92% dead, *N. meningitidis* HP-F AgNCs treated 76.49% dead, *S. pneumoniae* HP AgNCs treated 38% dead, and *S. pneumoniae* HP-F AgNCs treated 61.09% dead) (Figures 3 and S3–4). HP and HP-F AgNCs displayed comparable or improved activity to that of standard antibiotics. Both constructs displayed stronger activity against *N. meningitidis* compared to *S. pneumoniae*. Notably, the HP-F AgNCs enhanced antimicrobial activity against *S. pneumoniae* compared to the HP AgNCs supporting that HP arrangement on fibers can augment bactericidal activity (Figure 3). Together, these findings demonstrate that both constructs exert broad-spectrum antimicrobial activity against two major causative agents of bacterial meningitis, with effective inhibition achieved at low silver concentrations.

*N. meningitidis* and *S. pneumoniae* are primarily extracellular pathogens that initiate damaging neuroinflammatory responses during infection of resident glial cells within the central nervous system.^29,30^ To investigate the potential of employing HP and HP-F AgNCs as antimicrobials in the context of bacterial infection, human microglia cells were untreated or treated with constructs either prophylactically or therapeutically relative to infection with *N. meningitidis* or *S. pneumoniae*. Importantly, we observed no toxic effects of our constructs on human microglia, as shown by an MTS assay (Figure 4A, D, G, and J). Moreover, the constructs did not trigger any inflammatory response in microglia, as evidenced by the absence of IL-6 production during both prophylactic (Figure 4B and H) and therapeutic (Figure 4E and K) treatments. As anticipated, due to the inflammatory potential of *N. meningitidis* and *S. pneumoniae*, infection led to a significant increase in IL-6 production by human microglia (Figure 4B, E, H, and K). Notably, treatment with both constructs resulted in reduced IL-6 production following infection with both *N. meningitidis* (Figure 4B and E) and *S. pneumoniae* (Figure 4H and K). Excitingly, both prophylactic and therapeutic treatments led to a significant reduction in the bacterial viability of *N. meningitidis* and *S. pneumoniae* in infected microglia (Figure 4C, F, I, and L), with prophylactic treatment being more effective (Figure 4C and F). While both constructs were highly effective against planktonic *S. pneumoniae* (Figure 2C–D), we see that there is a reduced ability to restrict the bacterial burden in the context of infection (Figure 4I and L). In contrast, our data examining *N. meningitidis* (Figure 4C and F) infection demonstrated a 7-log reduction with HP and HP-F AgNC prophylactic treatment and a 3- to 7- log reduction with HP and HP-F AgNC therapeutic treatment, respectively. The observed differences in antimicrobial activity in the context of *N. meningitidis* and *S. pneumoniae* infection may reflect the distinct mechanisms of pathogenesis such as attachment or internalization for these bacterial species. Importantly, these findings demonstrate that AgNC formulations have a strong potential as antimicrobials agents capable of limiting bacterial burden and mitigating neuroinflammatory responses during bacterial meningitis.

This study establishes DNA-templated and stabilized AgNCs as a novel antimicrobial platform with a potential therapeutic relevance for bacterial meningitis. Both the HP and HP-F AgNCs exhibited stable physicochemical properties and red fluorescence suitable for bioimaging applications. Excitingly, both constructs displayed potent antimicrobial activity against *N. meningitidis* and *S. pneumoniae*, significantly reducing bacterial growth and viability while also attenuating infection-induced inflammatory responses in human microglia without inducing cytotoxicity. Notably, *N. meningitidis* exhibited greater sensitivity to treatments compared to *S. pneumoniae*, with lower silver concentrations sufficient to impair growth and reduce viability in planktonic cultures. This enhanced susceptibility of *N. meningitidis* is consistent with increased AgNC-induced membrane disruption, supporting membrane destabilization as a mechanism of antimicrobial activity. Our findings highlight the potential of biocompatible nanoscale silver formulations to function as antimicrobial agents for managing bacterial meningitis. Future studies will aim to further elucidate the mechanisms of bacterial inhibition and optimize DNA-AgNC design to enhance the selectivity and therapeutic efficacy against antimicrobial-resistant infections.

## Supplementary Material

SI

The Supporting Information is available free of charge at https://pubs.acs.org/doi/10.1021/acsabm.5c02143.

Methodology details and raw data (PDF)

## Figures and Tables

**Figure 1.**
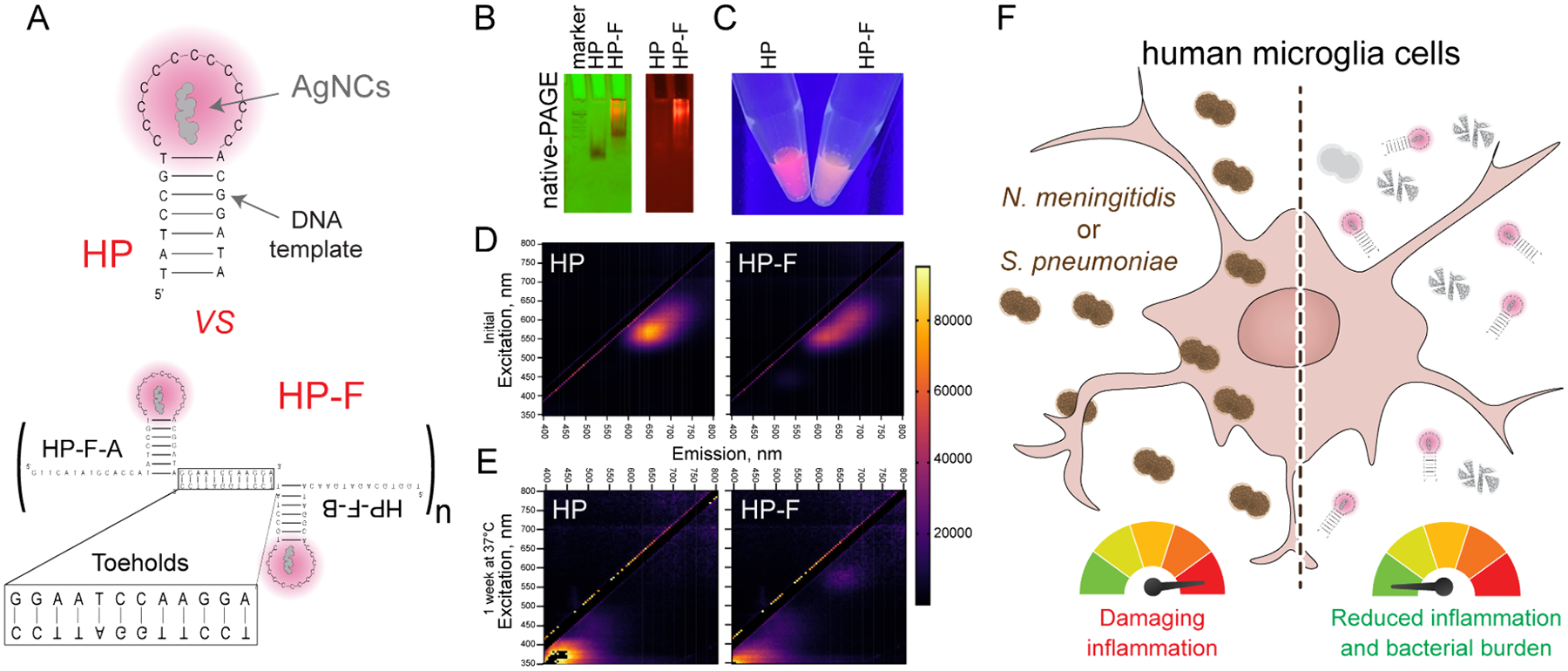
Physicochemical characterization of AgNCs templated either on a single DNA hairpin (HP) or on self-assembling multimeric hairpin fibrous structures (HP-F). (A) Schematic representation of the template sequences used to stabilize AgNCs. (B) 8% native-PAGE run at 250 V for 15 min and imaged using ChemiDoc. (Left) Multichannel image with ethidium bromide staining (green) and fluorescence imaging at 525 nm (red). (Right) 8% native PAGE evaluating fluorescence at 525 nm. (C) Fluorescence of AgNCs at 325 *μ*M silver when excited with 340 nm UV light. (D) Excitation–emission matrices of AgNCs at 130 *μ*M silver, highlighting the difference in fluorescence between the HP and HP-F AgNCs. Excitation spectra from 350 to 800 nm and emission spectra from 400 to 800 nm. (E) Excitation–emission matrices of AgNCs at 130 *μ*M silver after 1 week at 37 °C. (F) Delivery of HP and HP-F AgNCs to glial cells can restrict bacterial burden and consequently reduce damaging inflammatory responses.

**Figure 2.**
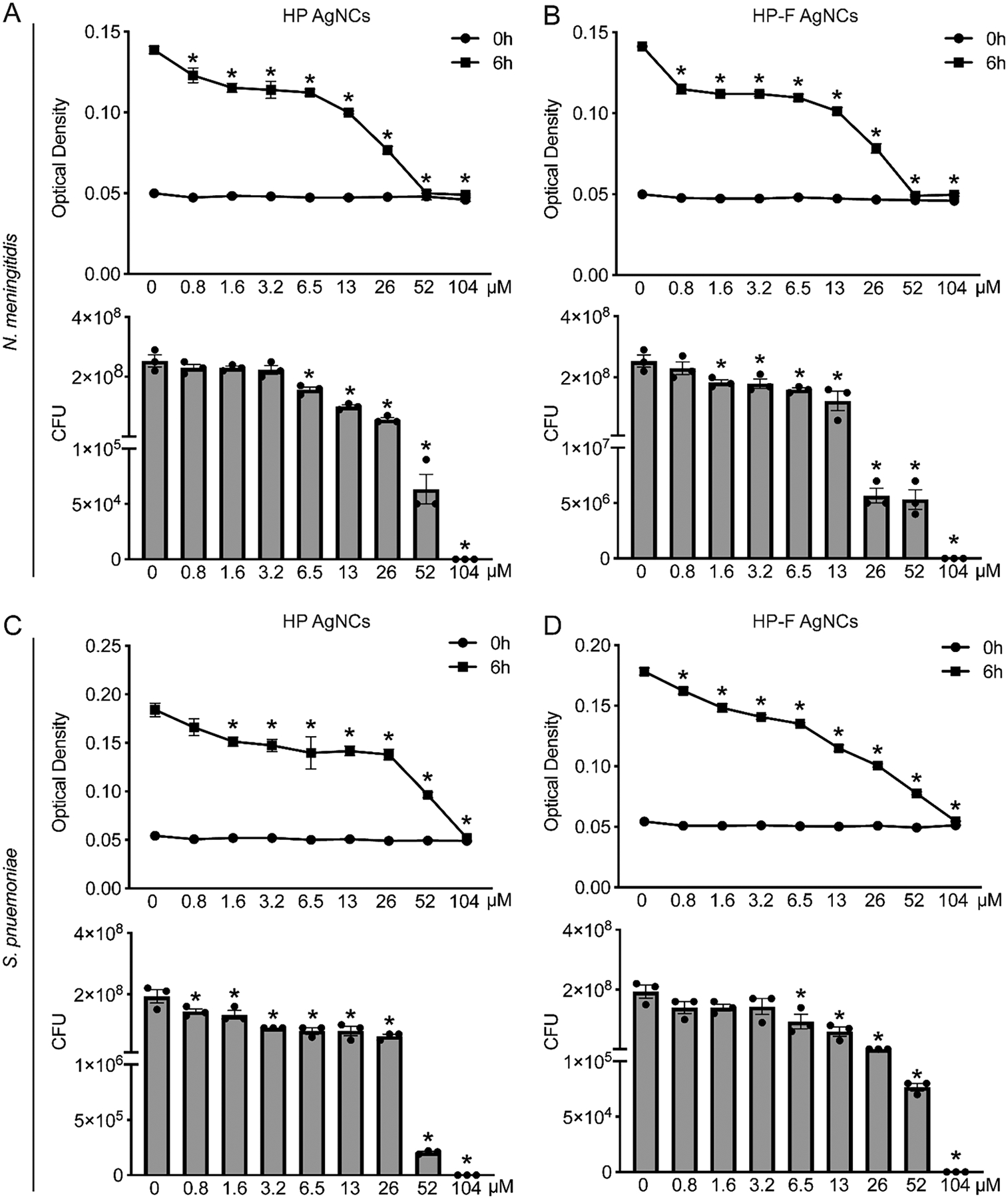
HP and HP-F AgNCs exhibit antimicrobial activity against *N. meningitidis* and *S. pneumoniae*. *N. meningitidis* and *S. pneumoniae* were untreated or incubated with the indicated silver concentrations of AgNCs. (A) Top: Optical density (OD) measurements of *N. meningitidis* at 6 h following incubation with HP AgNCs. Bottom: Colony-forming units (CFUs) of *N. meningitidis* at 6 h postincubation with HP AgNCs. (B) Top: OD measurements of *N. meningitidis* at 6 h following incubation with HP-F AgNCs. Bottom: CFUs of *N. meningitidis* at 6 h postincubation with HP-F AgNCs. (C) Top: OD measurements of *S. pneumoniae* at 6 h following incubation with HP AgNCs. Bottom: CFUs of *S. pneumoniae* at 6 h postincubation with HP AgNCs. (D) Top: OD measurements of *S. pneumoniae* at 6 h following incubation with HP-F AgNCs. Bottom: CFUs of *S. pneumoniae* at 6 h postincubation with HP-F AgNCs. Asterisks indicate a statistically significant difference compared to untreated bacterial cells. *x*-axis shows concentration of silver where 13 *μ*M silver corresponds to 1 *μ*M HP or 0.5 *μ*M HP-F DNA (mean ± SEM, *n* = 3; one-way ANOVA, *P* value < 0.05).

**Figure 3.**
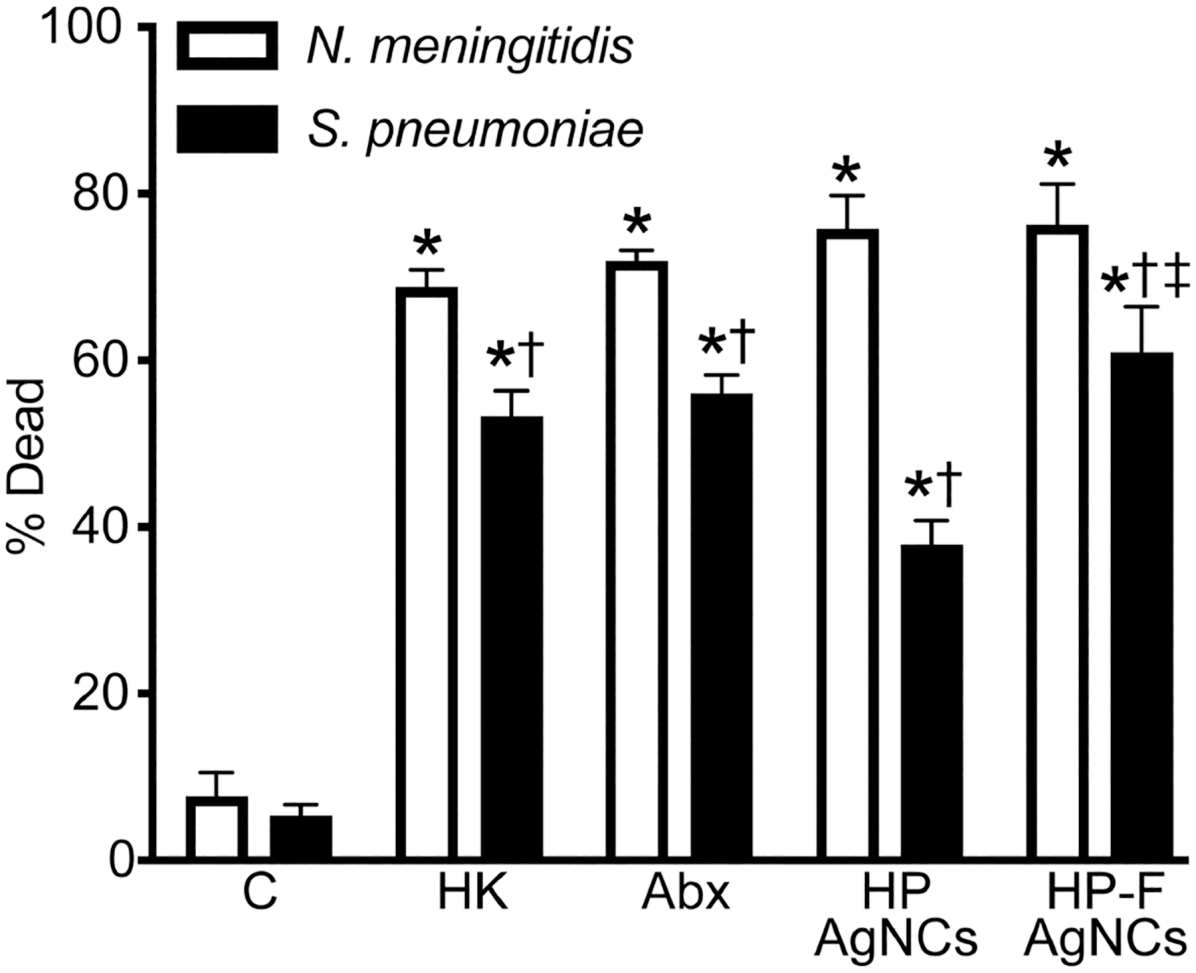
HP and HP-F AgNCs induce membrane damage in *N. meningitidis* and *S. pneumoniae*. *N. meningitidis* and *S. pneumoniae* were untreated (noted as C), heat-killed at 65 °C for 30 min (noted as HK), or treated for 3 h with the antibiotic, ceftriaxone (noted as Abx; 5 *μ*g/mL), 1 *μ*M HP AgNCs (13 *μ*M silver), or 0.5 *μ*M HP-F AgNCs (13 *μ*M silver). Following treatment, bacteria were stained with SYTOX Red Dead Cell Stain (5 nM) and analyzed by flow cytometry to assess membrane permeability. The percentage of dead cells was quantified as SYTOX-positive events and calculated using Overton subtraction relative to the untreated control. Asterisks indicate a statistically significant increase compared to the corresponding untreated control. Daggers represent a significant reduction between *N. meningitidis* and *S. pneumoniae*. Double daggers indicate a significant increase between *S. pneumoniae* treated with the HP and HP-F AgNCs (mean ± SEM, *n* = 3; two-way ANOVA, *P* value < 0.05).

**Figure 4.**
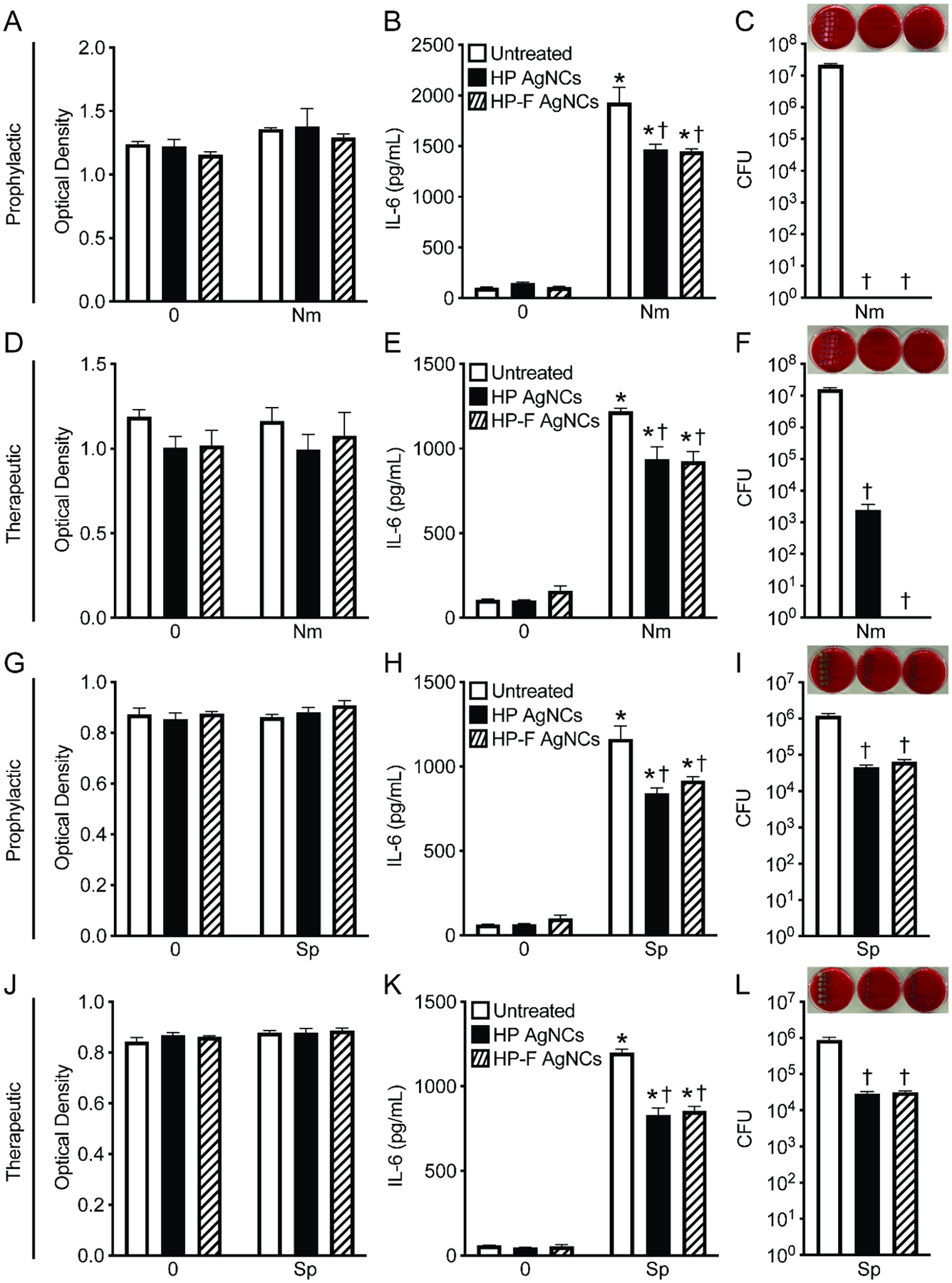
HP and HP-F AgNCs reduce inflammatory responses to and bacterial burden of *N. meningitidis* and *S. pneumoniae* during infection. Human microglia were untreated or treated with 6.5 *μ*M of silver constructs for 2 h either prophylactically or therapeutically relative to infection with *N. meningitidis* or *S. pneumoniae* (MOI 50:1) for 2 h. (A and G) Cell viability of human microglia was assessed with MTS assay at 6 h postinfection in the absence or presence of prophylactic constructs and *N. meningitidis* (A) or *S. pneumoniae* (G) challenge. (B and H) IL-6 production by human microglia at 6 h postinfection in the absence or presence of prophylactic constructs and *N. meningitidis* (B) or *S. pneumoniae* (H) challenge. (C and I) CFUs of *N. meningitidis* (C) or *S. pneumoniae* (I) at 6 h postinfection from human microglia untreated or treated prophylactically with constructs. (D and J) Cell viability of human microglia assessed with MTS assay at 6 h postinfection in the absence or presence of therapeutic constructs and *N. meningitidis* (D) or *S. pneumoniae* (J) challenge. (E and K) IL-6 production by human microglia at 6 h postinfection in the absence or presence of therapeutic constructs and *N. meningitidis* (E) or *S. pneumoniae* (K) challenge. (F and L) CFUs of *N. meningitidis* (F) or *S. pneumoniae* (L) at 6 h postinfection from human microglia untreated or treated therapeutically with constructs. Asterisks indicate a statistically significant increase compared to the corresponding uninfected control. Daggers represent a significant reduction compared to the untreated control (Mean ± SEM, *n* = 3; two-way ANOVA or Student’s *t* test, *P* value < 0.05).

## ABBREVIATIONS

AMR: antimicrobial resistance
AST: antimicrobial susceptibility testing
CNS: central nervous system
CFUs: colony-forming units
hH*μ*: human microglia
HP: single hairpin
AgNCs: DNA-templated silver nanoclusters
ELISA: enzyme-linked immunosorbent assay
HP-F: fibrous multimeric structure
Nm or *N. meningitidis*: *Neisseria meningitidis*
OD: optical density
PAGE: polyacrylamide gel electrophoresis
SEM: standard error of the mean
Sp or *S. pneumoniae*: *Streptococcus pneumoniae*
