# Supplementary material for: DNA-Templated Silver Nanoclusters Demonstrate Potent Antimicrobial Activity Against the Clinically Relevant Pathogens, Neisseria meningitidis and Streptococcus pneumoniae
Source: ACS Appl Bio Mater. Author manuscript; Available in PMC 2026 Mar 15. (PMC12958332; doi:10.1021/acsabm.5c02143)
Supplement: SI [file NIHMS2151846-supplement-SI.pdf]

Supporting Information For

DNA-templated Silver Nanoclusters Demonstrate Potent Antimicrobial Activity Against the Clinically Relevant Pathogens, *Neisseria meningitidis* and *Streptococcus pneumoniae*.

Krishna J. Majithia[1#], Elizabeth Skelly[2#], Kirill A. Afonin[2*], M. Brittany Johnson[1*]

[1]Department of Biological Sciences, University of North Carolina at Charlotte, Charlotte, NC, 28223 USA

[2]Chemistry and Nanoscale Science Program, Department of Chemistry, University of North Carolina at Charlotte, Charlotte, NC, 28223, USA

# These authors contributed equally to this project.

*- correspondence to mjohn398@charlotte.edu and kafonin@charlotte.edu

SUPPLEMENTAL FIGURES

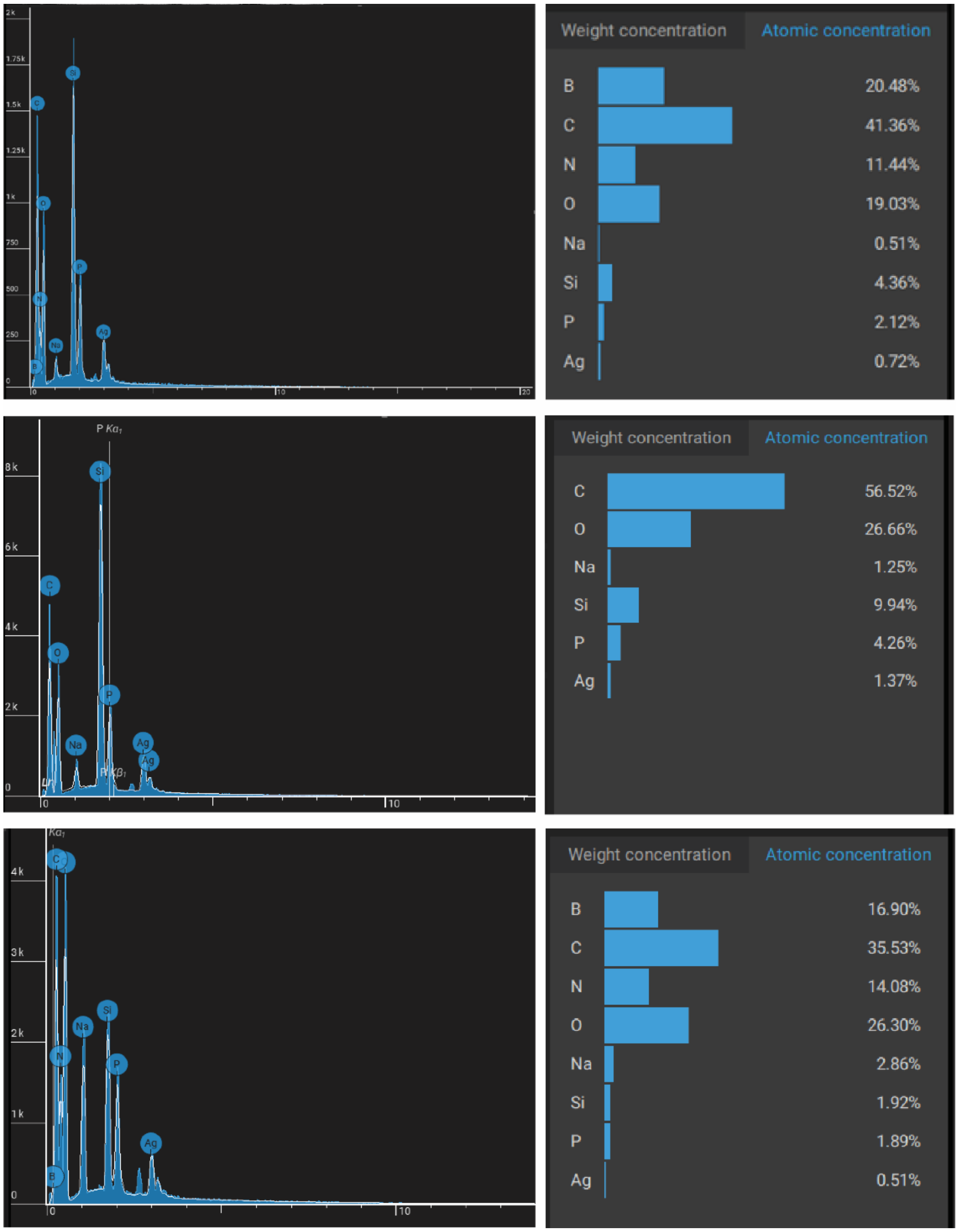

Figure S1: Raw data of Energy Dispersive X-Ray Spectroscopy (EDS) of HP AgNCs with the atomic concentration of each element in percent (n=3). It was calculated from these percentages that 8.59 ± 0.64 atoms of silver are on each HP structure.

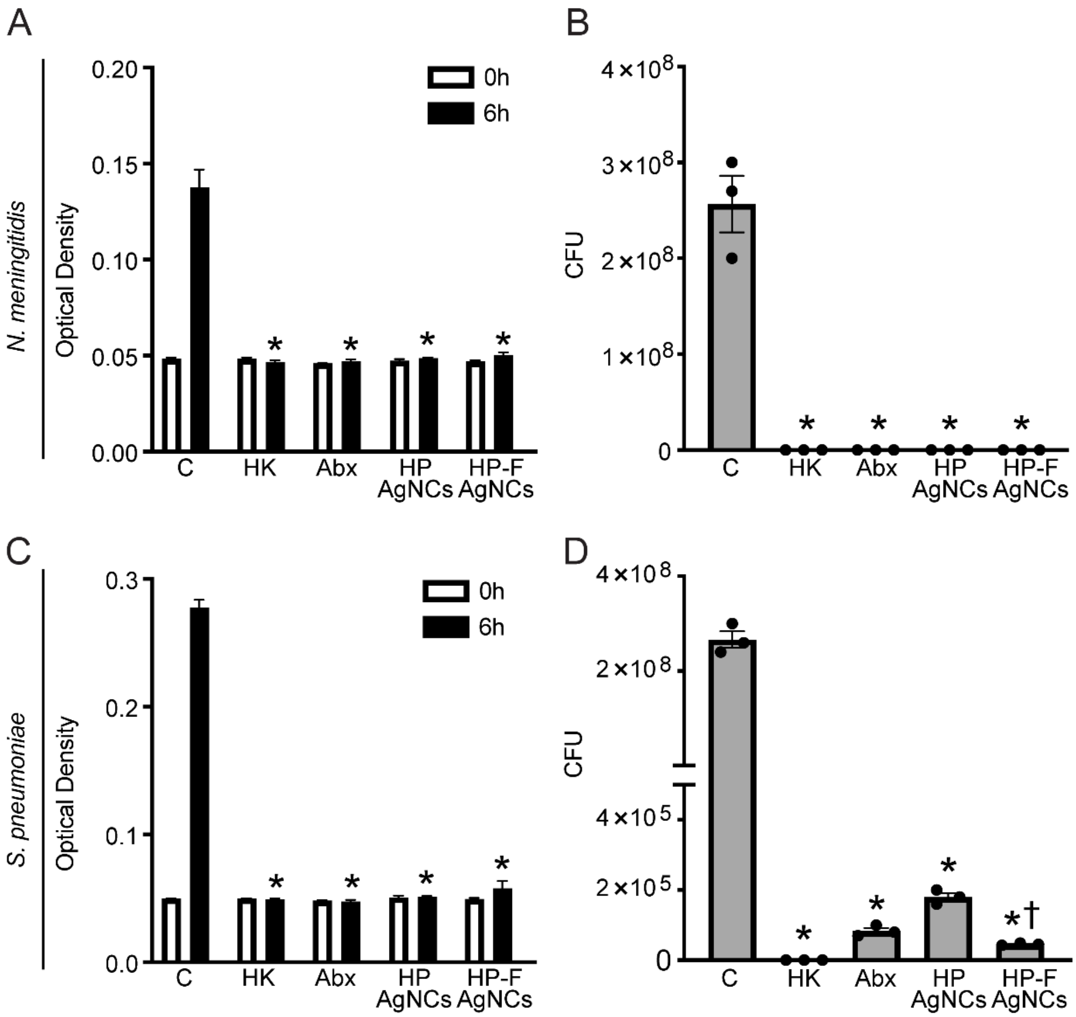

Figure S2: HP and HP-F AgNCs retain antimicrobial activity following prolonged storage at 37ºC. HP and HP-F AgNCs were stored at 37ºC for seven days prior to incubation with *N. meningitidis* or *S. pneumoniae.* (A) Optical density (OD) measurements of *N. meningitidis* untreated noted as C, heat-killed at 65ºC for 30 minutes noted as HK, or treated with the antibiotic, ceftriaxone (Abx; 5 µg/mL), HP AgNCs (104 µM silver) or HP-F AgNCs (104 µM silver) at 6h following incubation. (B) Colony-forming units (CFUs) of *N. meningitidis* at 6h post-incubation. (C) OD measurements of *S. pneumoniae* untreated (C), heat-killed at 65ºC for 30 minutes (HK), or treated with the antibiotic, ceftriaxone (Abx; 5 µg/mL), HP AgNCs (104 µM silver) or HP-F AgNCs (104 µM silver) at 6h following incubation. (D) CFUs of *S. pneumoniae* at 6h post-incubation. Asterisks indicate a statistically significant difference compared to untreated bacterial cells. Daggers indicate a significant reduction between HP and HP-F. (Mean ± SEM, n=3, one-way ANOVA, P value < 0.05).

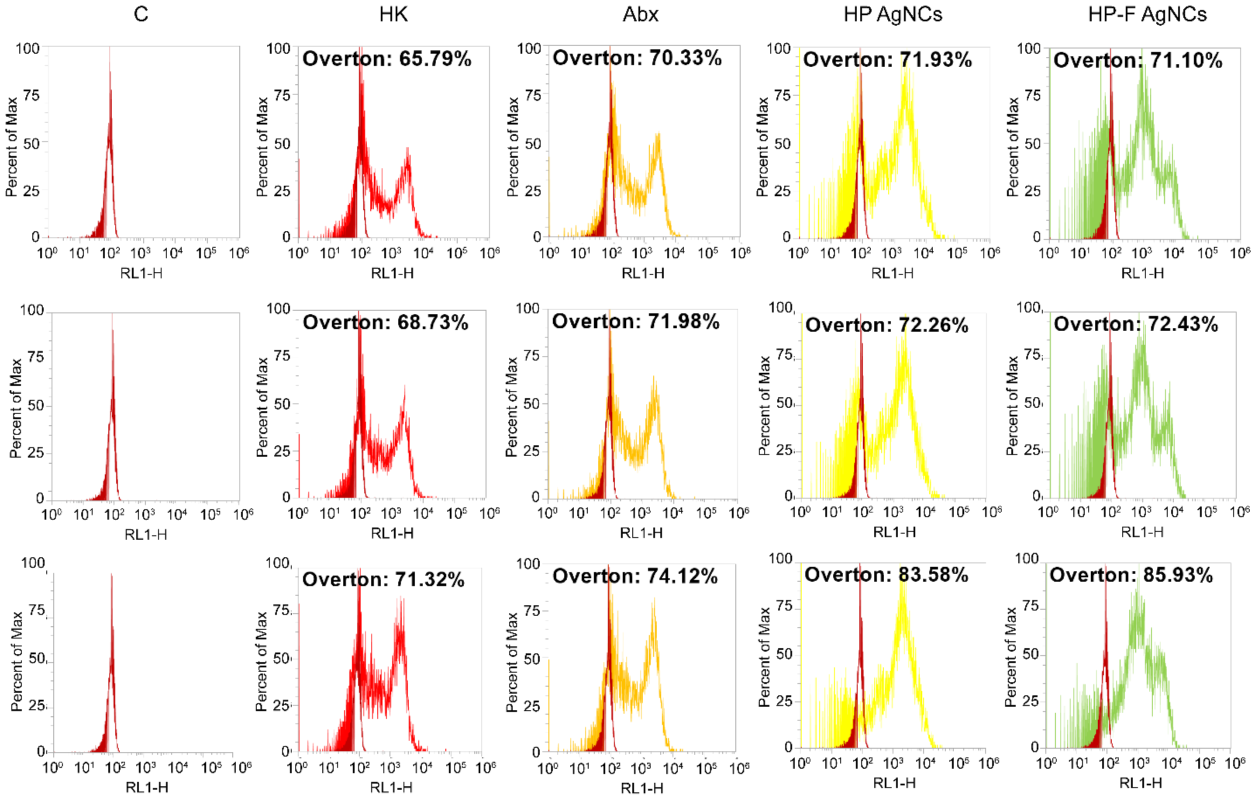

Figure S3: HP and HP-F AgNCs alter membrane integrity of *N. meningitidis.* *N. meningitidis* was untreated noted as C, heat-killed at 65ºC for 30 minutes noted as HK, or treated for 3h with the antibiotic, ceftriaxone (Abx; 5 µg/mL), HP AgNCs (13 µM silver) or HP-F AgNCs (13 µM silver). Following treatment, bacteria were stained with SYTOX Red Dead Cell Stain (5 nM) and analyzed by flow cytometry to assess membrane permeability. Representative fluorescence intensity histograms from n=3 experiments are shown. The percentage of dead cells was quantified as SYTOX-positive events and calculated using Overton subtraction relative to the untreated control.

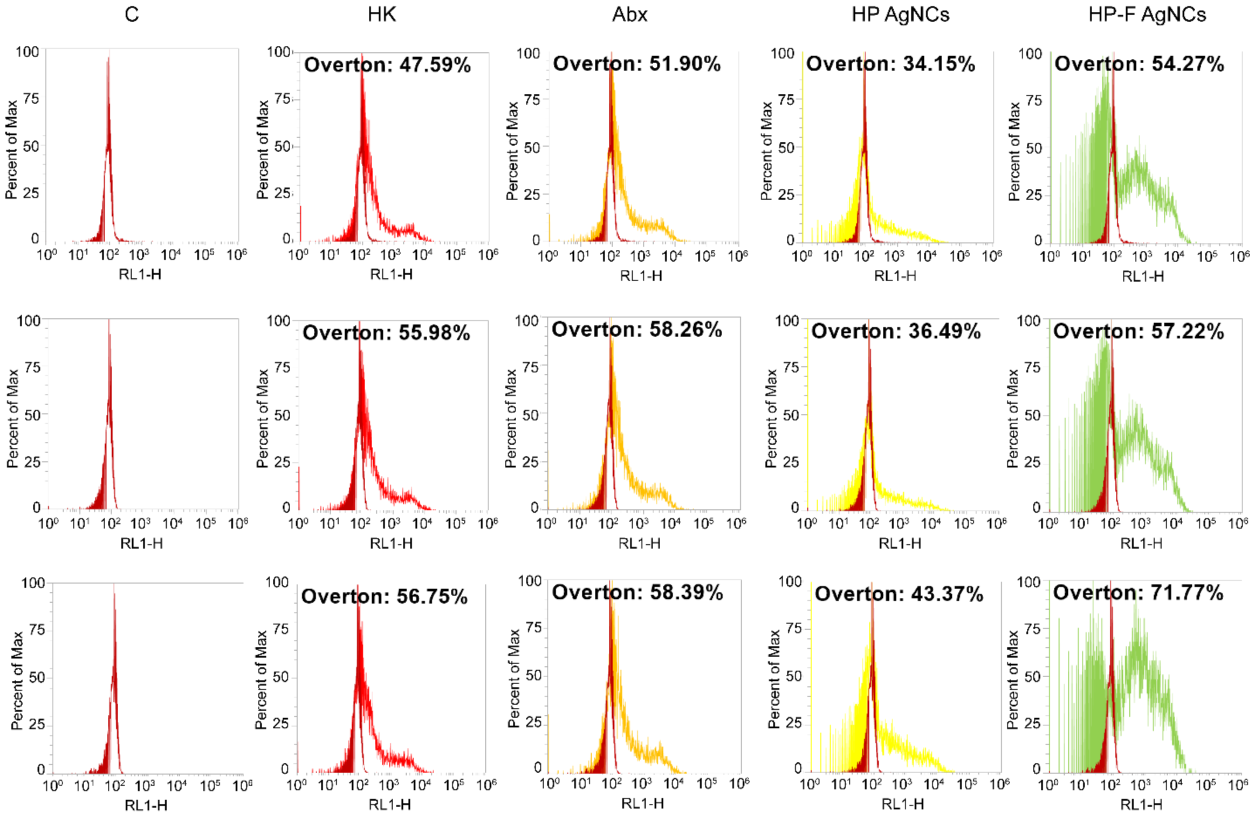

Figure S4: HP and HP-F alter membrane integrity of *S. pneumoniae.* *S. pneumoniae* was untreated noted as C, heat-killed at 65ºC for 30 minutes noted as HK, or treated for 3h with the antibiotic, ceftriaxone (Abx; 5 µg/mL), HP AgNCs (13 µM silver) or HP-F AgNCs (13 µM silver). Following treatment, bacteria were stained with SYTOX Red Dead Cell Stain (5 nM) and analyzed by flow cytometry to assess membrane permeability. Representative fluorescence intensity histograms from n=3 experiments are shown. The percentage of dead cells was quantified as SYTOX-positive events and calculated using Overton subtraction relative to the untreated control.

SEQUENCES USED IN THIS PROJECT

HP sequence: 5’-TATCCGTCCCCCCCCCCCCCACGGATA

HP-F sequences:

HP-F-A: 5’-GTTCATCTGCACCAACGGATACCCCCCCCCCCCCTATCCGTGGAATCCAAGGA

HP-F-B: 5’-TGGTGCAGATGAACACGGATACCCCCCCCCCCCCTATCCGTTCCTTGGATTCC

MATERIALS & METHODS

Motivation behind single stranded (ss)DNA sequences with cytosine hairpin loops

Silver ions bind to all 4 nucleotides (adenine, thymine, cytosine, and guanine) but have the highest affinity for cytosines, binding specifically to the nitrogen on the 3rd position in the pyrimidine structure[21]. While silver has a slightly lower but similar binding affinity for guanine, single stranded cytosine regions were chosen to avoid secondary structure such as G-quadruplexes. Furthermore, locally concentrating silver in one area in the DNA template allows for silver-silver bonds to form, increasing the stability of the clusters[25].

AgNCs synthesis

DNA scaffolds were purchased from Integrated DNA Technologies (IDT, Inc.). All DNA was purified using 8 M urea denaturing polyacrylamide gel electrophoresis (urea PAGE) and suspended in endotoxin-free double deionized (ddi)H_2O to ensure sterility of samples, as detailed previously when working with a variety of nucleic acids[24]. Synthesis of AgNCs followed our previously published work[28]. Briefly, HP AgNCs samples were synthesized one-pot where DNA (final concentration of 25 µM) was combined with $AgNO_3$ (final concentration of 325 µM), 20 mM NH_4OAc (final concentration of 4 mM), and endotoxin-free ddiH_2O. From there, the samples were briefly vortexed and centrifuged, then placed at 95°C for 2 minutes and immediately snap-cooled at 4°C for 20 minutes to form HPs. $NaBH_4$ was freshly diluted to 10 mM with cold ddiH_2O and added to the sample post incubation (final concentration of 325 µM). Alternatively, each DNA strand for HP-F AgNCs (Fiber-A and Fiber-B, final concentration of 12.5 µM) were separately incubated with endotoxin-free ddiH_2O at 95°C for 5 minutes. From there, both DNA strands were added to a single tube with $AgNO_3$ (final concentration of 325 µM) and 20 mM NH_4OAc (final concentration of 4 mM). The samples were then incubated at 25°C for 20 minutes to allow for HP-F assembly. $NaBH_4$ was freshly diluted to 10 mM with cold ddiH_2O and

added to the sample post incubation (final concentration of 325 µM). Following the addition of $NaBH_4$, the samples were kept in 4°C in the dark for at least 16 hours before use. Samples were run on 8% native polyacrylamide gel electrophoresis (PAGE, 19:1 acrylamide:bisacrylamide) for 15 minutes at 250 volts. To purify HP and HP-F AgNCs from free silver and byproducts before antibacterial studies, samples were washed thrice by centrifugation at 12000 rcf for 15 minutes, with 20% final volume 4 mM NH_4OAc added prior to the second and third washes.

Excitation-Emission Spectroscopy (EEM)

100 µL of HP and HP-F AgNCs at 130 µM silver were loaded into a 96-well black walled plate and the 3D EEM was measured in a Tecan Spark microplate reader. Excitation data was measured over a range of 350 to 800 nm with a manual gain of 150, a 5 nm step size between measurements with a 5 nm bandwidth. Emission data was measured over a range of 400 to 800 nm with the same gain, step size, and bandwidth. The EEM was plotted using GraphPad Prism.

Energy Dispersive X-Ray Spectroscopy (EDS)

HP AgNCs were prepared and washed following the above protocol. 5 µL of HP AgNCs at 130 µM silver dispersed on a clean silica wafer and dried at 50ºC for 1 minute. This was repeated on the same spot on the wafer until 25 µL were dried on the wafer. Thermo-Fisher Phenom XL desktop SEM was used to perform EDS. The percentage of all atoms was found. From the percentage, the number of silver atoms was calculated based on the known number of phosphates per structure. This was repeated 3 times with the average and standard deviation found.

Culture of human microglia cell line

The human microglial cell line (hμglia) was generously provided by Dr. Jonathan Karn (Case Western Reserve University). This line was derived from primary human cells that were transformed with lentiviral vectors encoding SV40 T antigen and human telomerase reverse transcriptase. Comprehensive characterization and classification of these cells have been previously described[29]. Human microglia display microglia-like morphology, evidenced by the expression of surface markers such as CDB11, TGFBR, and P2RY12, along with phagocytic activity. Cells were maintained in Dulbecco's Modified Eagle Medium (DMEM) supplemented with 5% fetal bovine serum (FBS) and 100 U/ml penicillin-100 μg/ml streptomycin at 37ºC in a humidified atmosphere with 5% CO_2.

N. meningitidis **and** ***S. pneumoniae*** **propagation**

Neisseria meningitidis strain MC58 (ATCC BAA-335) and *Streptococcus pneumoniae* strain CS109 (ATCC 51915) were grown on Columbia agar plates supplemented with 5% defibrinated sheep blood overnight followed by culturing in Columbia broth at 37ºC and 5% CO_2 overnight. The number of colony-forming units (CFU) was determined using a Genespec3 spectrophotometer as previously described (MiraiBio Inc.)[29, 30].

Bacterial viability

N. meningitidis and *S. pneumoniae* were seeded at a density of 1 x 10^6 CFU per well in a 96-well plate. Cells were either left untreated or exposed to various concentrations of HP and HP-F AgNCs in Columbia broth for 6h on an orbital shaker at 37ºC and 5% $CO_{2.}$ Following incubation, serial dilutions were prepared and plated on Columbia agar plates overnight. The number of viable colonies was determined by colony counting.

Bacterial infection of human microglia

Human microglia were seeded at a density of 5 x 10^4 cells per well and infected with *N. meningitidis* or *S. pneumoniae* at a multiplicity of infection (MOI) of 50:1 bacteria per host cell in antibiotic-free medium for 2h, as previously described[25]. Following infection, the medium was replaced with fresh medium without antibiotics. At 6h post-infection, cell supernatants were collected for analysis. Additionally, microglia were lysed using saponin and both intracellular and extracellular CFUs were plated on Columbia agar plates overnight. The number of viable colonies were assessed by colony counting.

AgNC treatment of human microglia

Human microglia were seeded at density of 5 x 10^4 cells per well and extracellularly treated with HP (0.5 μM DNA, 6.5 μM Ag) and HP-F AgNCs (0.25 μM DNA, 6.5 μM Ag) for 2h in antibiotic-free medium either prior to or following infection with *N. meningitidis* or *S. pneumoniae* at a multiplicity of infection (MOI) of 50:1 bacteria per host cell as previously described. At 6h post-infection, cell supernatants were collected for analysis. Additionally, microglia were lysed using saponin and both intracellular and extracellular CFUs were plated on Columbia agar plates overnight. The number of viable colonies were assessed by colony counting.

Enzyme-linked immunosorbent assays

Specific capture enzyme-linked immunosorbent assays (ELISAs) were performed to quantify the production of interleukin-6 (IL-6) by human microglia in response to infection and treatment with HP and HP-F AgNCs. Concentrations of IL-6 were measured using commercially available antibody pairs (BD Biosciences; 554543, 554546) and according to established protocols[29, 30]. Standard curves were generated with recombinant proteins and protein concentrations in cell supernatants were determined by comparing sample absorbance values to the appropriate standard curve.

MTS cell viability assay

Human microglia were seeded at density of 5 x 10^4 cells per well and extracellularly treated with HP (0.5 µM DNA, 6.5 µM Ag) and HP-F AgNCs (0.25 µM DNA, 6.5 µM Ag) for 2h in antibiotic-free medium either prior to or following infection with *N. meningitidis* or *S. pneumoniae* at a multiplicity of infection (MOI) of 50:1 bacteria per host cell as previously described. At 6h post-infection, MTS reagent was added to cells at a dilution of 1:5 (Promega Corporation; G3580) and incubated for 1h at 37ºC and 5% CO_2. Following incubation, plates were analyzed at 490nm using Molecular Devices SpectraMax iD5 plate reader.

Flow cytometric analysis of bacterial membrane integrity

Membrane integrity of *N. meningitidis* and *S. pneumoniae* was assessed by flow cytometry using SYTOX Red Dead Cell Stain (Invitrogen). Bacteria were left untreated (control), heat-killed at 65 °C for 30 min, or treated with ceftriaxone (5 µg/mL), HP AgNCs (13 µM silver), or HP-F AgNCs (13 µM silver) for 3 h at 37 °C. Following treatment, bacterial cells were stained with SYTOX Red (5 nM) for 15 minutes at room temperature in the dark. Samples were then analyzed by flow cytometry (AttuneNXT Acoustic Focusing Cytometer, Thermo Fisher Scientific) using forward and side scatter parameters to identify the bacterial population and exclude debris. A total of 10,000 events were collected per sample. Untreated and unstained bacteria were used to establish baseline gating. Heat-killed bacteria were used as a positive control for SYTOX-positive events. Fluorescence intensity histograms were generated, and the percentage of dead cells was quantified as SYTOX-positive events using Overton subtraction relative to the untreated control.

Statistical analysis

Data are presented as the mean ± standard error of the mean (SEM). Statistical analyses were conducted using GraphPad Prism (GraphPad Software, La Jolla, CA, USA).

Depending on the experimental design, comparisons were made using Student's t-test, one-way analysis of variance (ANOVA) with Dunnett's post hoc test, or two-way ANOVA with Šídák's multiple comparisons test. A *P*-value < 0.05 was considered statistically significant.
